# Supplementary material for: Health-related quality of life and treatment satisfaction in Chronic Lymphocytic Leukemia (CLL) patients on ibrutinib compared to other CLL treatments in a real-world US cross sectional study
Source: PLoS One. 2022 Oct 6;17(10):e0270291. doi: 10.1371/journal.pone.0270291 (PMC9536620; doi:10.1371/journal.pone.0270291)
Supplement: Attachment — Submitted filename: Response to Reviewers.docx [file pone.0270291.s001.docx]

**Reviewer #1:**

1) The authors excluded patients who did not have at least 6 months of ibrutinib so may have missed patients who discontinued early or who initially experience side effects potentially biasing sample to favor improved HRQoL. Information on duration of treatment with ibrutinib should be provided

**RESPONSE: We added the reported duration of treatment for ibrutinib and a note in the limitations regarding the exclusion of patients who did not have at least 6 months of ibrutinib.**

2) For the patients in the control arm, there is no information on whether they were actively on treatment or had concluded treatment with chemoimmunotherapy or other finite therapy (the authors should describe the types of therapies patients in the control group had been treated with). This also could have confounded their HRQoL

**RESPONSE: We added the type of treatment therapies for the chemotherapy group and the number that reported not currently receiving treatment. Additionally, we included a statement in the Limitations section.**

3) The authors should provide more information on the clinical characteristics e.g., presence of 17p deletions and other high risk mutations, presence of CLL constitutional symptoms at baseline i.e, fatigue, nausea and weight loss. Could be added as a supplement if space is an issue

**RESPONSE: These were not gathered as the authors and recruiting institutions felt the reliability and recall bias would be too great since all data was collected via patient reporting.**

4) The difference in recruitment method is also a limitation. The ibrutinib patients were primarily recruited through treating physicians (and a small number – 23 – through specialty pharmacies), whereas the reference groups were recruited through a consumer market research panel. For the latter, how did the investigators confirm diagnosis and treatment history/eligibility for these patients. Perhaps those recruited by physicians would be more likely to report better HRQoL and greater satisfaction

**RESPONSE: We note that the different recruitment methods could have resulted in selection bias. The CLL Norming Study relied on patient’s report of disease and treatment. We have included this limitation in the discussion**

5) "This study evaluates the impact of ibrutinib on HRQoL in a real-world population to understand the impact of ibrutinib at different points in their real-world treatment journey." This conclusion is not valid given the cross-sectional design of the study

**RESPONSE: We added cross-sectional to the statement referenced. We feel this is a reasonable conclusion as patients were at different point of their disease and treatment journey at the time of participation.**

6) Rationale for instrument selection should be provided. There seems to be overlap in the FACT-Leu and FACIT instruments and may have redundant to administer both. Why didn't they administer the EORTC-QLQ-C30 used in the Ibrutinib clinical trials? How did the survey completion rates by phone compare with online?

**RESPONSE: Though this evaluation focuses on the CLL population, the Norming Study included groups of patients with other cancers (prostate, lung, multiple myeloma, other leukemias and lymphomas) and a general population group without cancer. Due to the inclusion of other cancer, the FACT-G was chosen as an instrument that could be administered to all cancer groups and the FACT-LeuS helped minimize the survey burden versus including the EORTC QLQ-C30 in addition to the FACT-G. This information and rationale was added to the manuscript.** **The survey completion rates were not provided by the recruiting partners.** **All patients that qualified and started the surveys were able to complete them regardless of mechanism (online or phone).**

**Reviewer #2:**

1. The authors have compared the HR-QoL in CLL patients from the Norming Study 64 were treatment naive (Tx naive), 36 were 1st line (1L),and 38 were in or had completed ≥2 lines (2L+) patients with CLL with CLL Ibrutinib Study included 1L and 2L+ ibrutinib patients with CLL treated for ≥6 months in which 118 patients (1L n= 88 and 2L+ n=30)

Though the differences in sample size were taken care of by using the Hedges g which takes care of different sample sizes, the patient groups are heterogenous. For example it is expected that there will be differences in QoL for CLL patients on 1L and 2L therapy, so comparison of Ibrutinib (1L+2L) versus CIT (1L+2L) needs to be done differently for 1L and 2L separately.

**RESPONSE: We have added a figure and text with the comparisons of Ibr 1L to Other 1L treatments and Ibr 2L+ to Other 2L+ treatments. We also added the note in the limitations that results are not adjusted for previous treatments or duration on current treatment.**

2. The authors have only provided effect sizes for comparison. Could they also provide statistical p values and clinical significance values for each of the domain comparisons? Fig 1-6 don't make sense without these values.

**RESPONSE: All the p-values were significant for the comparisons. We added a statement in the footnotes for each figure.**

3. It is mentioned that patients on ibrutinib had to be on therapy for at least 6 months before inclusion for QoL assessment. What was the median time on therapy for ibrutinib patients since there is 50% discontinuation rates reported in literature with time. The same time period for patients on CIT needs to be mentioned. At what time on/ after CIT were patients included. Since patients on CIT are likely to report improvement in QoL after completion of time limited therapy.

**RESPONSE: We added the type of treatment therapies for the CLL Norming Study groups and the number that reported not currently receiving treatment. Unfortunately, the time on therapy for the CLL Norming Study was largely missing and not able to be reported. The time on ibrutinib was collected as a categorical variable and has been added to the manuscript.**

4. The prognostic index or IgHV/Tp53 mutation status if available needs to be mentioned in both cohorts, since patients with either positivity on CIT don't do well.

**RESPONSE: We have added the following paragraph to address this point “Although it is known that IGHV and TP53 mutation status affect response to CIT, which could affect HRQoL reports, it is also known that the rate of testing in routine clinical practice is less than optimal. In a recent study presented at ASH 2020, Mato and colleagues found that TP53 mutation testing was performed in only 11% (n=162) of pts and IGHV mutational status testing was performed in 12%** [1]**. Since all information was patient-reported and there was no option to confirm the reliability of the prognostic marker testing, the authors did not obtain this information given potential recall bias.”**
1.	Mato AR, Barrientos JC, Sharman JP, et al. Real-World Prognostic Biomarker Testing, Treatment Patterns and Dosing Among 1461 Patients (pts) with Chronic Lymphocytic Leukemia/Small Lymphocytic Lymphoma (CLL/SLL) from the informCLL Prospective Observational. American Society of Hematology 62nd Annual Meeting and Exposition. Abstract 547.

5. Please add data on patients screened, answered, missing data

**RESPONSE: We do not have data on the number of patients screened. For the patients that completed the survey, there was no missing data.** 6. The reasons why patients on ibrutinib appear to be doing well than TN patients on observation needs explanation.

**RESPONSE: The discussion includes references with similar findings and findings related to impaired HRQoL in patients on active surveillance. Added a statement to regarding the possible reasonings behind the impairments in HRQoL of patients on active surveillance.**
