# Supplementary material for: Health-related quality of life and treatment satisfaction in Chronic Lymphocytic Leukemia (CLL) patients on ibrutinib compared to other CLL treatments in a real-world US cross sectional study
Source: PLoS One. 2022 Oct 6;17(10):e0270291. doi: 10.1371/journal.pone.0270291 (PMC9536620; doi:10.1371/journal.pone.0270291)
Supplement: Attachment — Submitted filename: PLOS One_PONE-D-21-02744_Response to Reviewer_11April2022.docx [file pone.0270291.s002.docx]

Response to Reviewers

22 April 2022

EPI-Q, Inc.

915 Harger Road, Suite 350 Oak Brook, IL 60523-1494

630.570.5505 ext. 410

Dear Editors,

We had previously submitted our manuscript entitled “Health-related quality of life and treatment satisfaction in Chronic Lymphocytic Leukemia (CLL) patients on ibrutinib compared to other CLL treatments in a real-world US cross sectional study”, PONE-D-21-02744.

We have responded to the reviewers and editors comments and are now resubmitting. We appreciate the thoughtful and thorough review and hope to have a successful response to the updated manuscript.

Below is a point by point summary of the changes made in response to peer review. We appreciate the reviewers’ comments and believe these edits strengthen the manuscript.

Sincerely,

Kathleen L Deering

Kathleen L Deering

Corresponding author

**Journal Requirements:**

When submitting your revision, we need you to address these additional requirements.

1. Please ensure that your manuscript meets PLOS ONE's style requirements, including those for file naming. The PLOS ONE style templates can be found at
   [https://journals.plos.org/plosone/s/file?id=wjVg/PLOSOne_formatting_sample_main_body.pdf](https://nam10.safelinks.protection.outlook.com/?url=https%3A%2F%2Fjournals.plos.org%2Fplosone%2Fs%2Ffile%3Fid%3DwjVg%2FPLOSOne_formatting_sample_main_body.pdf&data=04%7C01%7Ckatie.deering%40epi-q.com%7C9e26b677424b4148211e08d9aa9febdf%7C712244b46d0d433f9075d47b19b2dda5%7C0%7C0%7C637728426112816708%7CUnknown%7CTWFpbGZsb3d8eyJWIjoiMC4wLjAwMDAiLCJQIjoiV2luMzIiLCJBTiI6Ik1haWwiLCJXVCI6Mn0%3D%7C3000&sdata=XK5qZQk85Wvp8EmdqBiuBUfq6%2B0UAj%2FoBGoeBldWz5E%3D&reserved=0) and 
   [https://journals.plos.org/plosone/s/file?id=ba62/PLOSOne_formatting_sample_title_authors_affiliations.pdf](https://nam10.safelinks.protection.outlook.com/?url=https%3A%2F%2Fjournals.plos.org%2Fplosone%2Fs%2Ffile%3Fid%3Dba62%2FPLOSOne_formatting_sample_title_authors_affiliations.pdf&data=04%7C01%7Ckatie.deering%40epi-q.com%7C9e26b677424b4148211e08d9aa9febdf%7C712244b46d0d433f9075d47b19b2dda5%7C0%7C0%7C637728426112826671%7CUnknown%7CTWFpbGZsb3d8eyJWIjoiMC4wLjAwMDAiLCJQIjoiV2luMzIiLCJBTiI6Ik1haWwiLCJXVCI6Mn0%3D%7C3000&sdata=KcdHpZPg%2B%2FbmOousOPWiGWVbuxSzdkhRTGt%2B4Yy6rEs%3D&reserved=0)

**RESPONSE: We have reviewed and made revisions as necessary.**

2. Please amend your current ethics statement to address the following concerns:
   a) Did participants provide their written or verbal informed consent to participate in this study? 
   b) If consent was verbal, please explain i) why written consent was not obtained, ii) how you documented participant consent, and iii) whether the ethics committees/IRB approved this consent procedure.

**RESPONSE: We have added statements for why written consent was not obtained and noted that the procedure used was approved by the IRB.**

3. Thank you for stating the following in the Competing Interests section:

"MS was an employee of Janssen, and JT is currently an employee of Janssen. KLD and QH are employees of EPI-Q Inc., which received payment from Janssen
associated with the development and execution of this study. JCB has received
honoraria for consulting for Gilead, AstraZeneca, Janssen, Sandoz, Genentech, Bayer,
and Celgene. JCB has received research support from Gilead and
Pharmacyclics/AbbVie."

Please confirm that this does not alter your adherence to all PLOS ONE policies on sharing data and materials, by including the following statement: "This does not alter our adherence to  PLOS ONE policies on sharing data and materials.” (as detailed online in our guide for authors [http://journals.plos.org/plosone/s/competing-interests](https://nam10.safelinks.protection.outlook.com/?url=http%3A%2F%2Fjournals.plos.org%2Fplosone%2Fs%2Fcompeting-interests&data=04%7C01%7Ckatie.deering%40epi-q.com%7C9e26b677424b4148211e08d9aa9febdf%7C712244b46d0d433f9075d47b19b2dda5%7C0%7C0%7C637728426112826671%7CUnknown%7CTWFpbGZsb3d8eyJWIjoiMC4wLjAwMDAiLCJQIjoiV2luMzIiLCJBTiI6Ik1haWwiLCJXVCI6Mn0%3D%7C3000&sdata=XhDbbiS7iVIKu%2F4rtQ%2F09FhnFABAAQTXeeNTOySpSEM%3D&reserved=0)).  If there are restrictions on sharing of data and/or materials, please state these. Please note that we cannot proceed with consideration of your article until this information has been declared.

Please include your updated Competing Interests statement in your cover letter; we will change the online submission form on your behalf.

**RESPONSE: Please update the Competing Interests statement with the following “This does not alter our adherence to PLOS ONE policies on sharing data and materials.”**

4. We note that you have indicated that data from this study are available upon request. PLOS only allows data to be available upon request if there are legal or ethical restrictions on sharing data publicly. For more information on unacceptable data access restrictions, please see [http://journals.plos.org/plosone/s/data-availability#loc-unacceptable-data-access-restrictions](https://nam10.safelinks.protection.outlook.com/?url=http%3A%2F%2Fjournals.plos.org%2Fplosone%2Fs%2Fdata-availability%23loc-unacceptable-data-access-restrictions&data=04%7C01%7Ckatie.deering%40epi-q.com%7C9e26b677424b4148211e08d9aa9febdf%7C712244b46d0d433f9075d47b19b2dda5%7C0%7C0%7C637728426112836664%7CUnknown%7CTWFpbGZsb3d8eyJWIjoiMC4wLjAwMDAiLCJQIjoiV2luMzIiLCJBTiI6Ik1haWwiLCJXVCI6Mn0%3D%7C3000&sdata=r1PW%2FJD1PYIDa0ymGApO42zDrPYH4BHS0sIaLasDEP8%3D&reserved=0).

In your revised cover letter, please address the following prompts:

a) If there are ethical or legal restrictions on sharing a de-identified data set, please explain them in detail (e.g., data contain potentially sensitive information, data are owned by a third-party organization, etc.) and who has imposed them (e.g., an ethics committee). Please also provide contact information for a data access committee, ethics committee, or other institutional body to which data requests may be sent.

b) If there are no restrictions, please upload the minimal anonymized data set necessary to replicate your study findings as either Supporting Information files or to a stable, public repository and provide us with the relevant URLs, DOIs, or accession numbers. For a list of acceptable repositories, please see [http://journals.plos.org/plosone/s/data-availability#loc-recommended-repositories](https://nam10.safelinks.protection.outlook.com/?url=http%3A%2F%2Fjournals.plos.org%2Fplosone%2Fs%2Fdata-availability%23loc-recommended-repositories&data=04%7C01%7Ckatie.deering%40epi-q.com%7C9e26b677424b4148211e08d9aa9febdf%7C712244b46d0d433f9075d47b19b2dda5%7C0%7C0%7C637728426112846580%7CUnknown%7CTWFpbGZsb3d8eyJWIjoiMC4wLjAwMDAiLCJQIjoiV2luMzIiLCJBTiI6Ik1haWwiLCJXVCI6Mn0%3D%7C3000&sdata=XrEk3sfVS%2FdBjD7z3P09zM7i%2BqGiIG7jiIP%2FaPHGFq0%3D&reserved=0).

We will update your Data Availability statement on your behalf to reflect the information you provide.

**RESPONSE: We have uploaded a minimal anonymized data set and it can be found at .**

**Reviewer #1:**

The authors have addressed all concerns.

**RESPONSE: Thank you for your thorough review and acceptance of our revisions.**

**Reviewer #2:**

Here Deering et al report analyze PRO among patients with CLL treated with ibrutinib or not. The data suggest that ibrutinib treatment is associated with improved QOL, even as compared with patients who are treatment-naïve.

1. The authors do not present statistical analysis of their data, ie comparison between

**RESPONSE: The study evaluated several populations and was not powered to detect differences among or between them. The authors did not feel it appropriate to generate statistical tests as that could lead to misinterpretation of the data.**

2. Table 1 – it would be helpful to summarize comorbidities in this Table, in addition to listing individual diseases Perhaps, a Charlson comorbidity score could be used to achieve that. Was the severity of comorbidities known?

**RESPONSE: Not all of the comorbidities in the Charlson comorbidity score were collected in the survey. The authors added the average number of comorbidities and the number and percentage of patients with 3+ comorbidities to try and better describe the burden.**

3. In connection with that, the Results show that patients in the Norming study had higher burden of comorbidities, despite being younger. Is there an explanation for that? Perhaps using an index like Charlson might help equalize the groups.

**RESPONSE: As described in the results, the IBR population had a higher comorbidity burden, not the Norming study. Within the Norming study population, the Naïve patients were older and had a higher comorbidity burden. The comorbidities that were higher in the Norming Study Naïve population than the Norming Study 1L or 2L+ populations were generally diseases that increase with age. Additionally, the Norming Study Naïve population was twice the size as the other two Norming Study populations. Thus, the differences in comorbidities may be related to the age difference (~10 years) or the population size. We have included this in the limitations.**

4. It is interesting that ibrutinib-treated patients reported improved QOL measures compared with treatment-naïve patients with CLL. The authors try to explain that by the requirement for therapy based on IWCLL criteria. However, it is presumed that the majority of CLL patients in the norming study are not symptomatic from CLL. However, they do have a higher burden of comorbidities. It is possible that comorbidities contribute to lower scores. This needs to be explained further/discussed as a limitation

**RESPONSE: This is a possibility and have included it in the limitations.**

5. Statistical significance should be presented on the graphs rather than be part of the legend. Indicate p<0.01 and p<0.05. Use of asterix is acceptable.

**RESPONSE: The authors feel that it is difficult to present the statistical significance on the graph. We felt an asterisk would not be able to distinguish that CLL Ibrutinib groups were not statistically significant compared to the CLL Norming Treatment Naïve group and brackets would make the graph very busy and hard to read. These p-values were requested by a past reviewer; however, the groups were not powered to detect differences and the authors do not want to over emphasize and risk misinterpretation of the data. If the reviewer has another suggestion, we are open to considering.**

6. The authors should discuss what are clinically meaningful improvement in these scales?

**RESPONSE: Clinically meaningful improvements are generally reserved for measuring improvements for an individual from one time period to another. We only evaluated a cross-sectional measurement for groups and did not feel it was appropriate to evaluate and include clinically meaningful improvements.**

7. There are multiple typos throughout the manuscript. Please check carefully. Also, please italicize gene names, such as IGHV and TP53.

**RESPONSE: Apologies for missing any typos following the last round of revisions. We have reviewed carefully and made corrections noted by the reviewer. Of note, some of the references used British English spellings in their titles so these are not corrected for American English.**
